# Genetic and environmental contributions to psychological resilience and coping

**DOI:** 10.12688/wellcomeopenres.13854.1

**Published:** 2018-02-15

**Authors:** Lauren B Navrady, Yanni Zeng, Toni-Kim Clarke, Mark J Adams, David M Howard, Ian J Deary, Andrew M McIntosh

**Affiliations:** 1 Division of Psychiatry, University of Edinburgh, Edinburgh , EH10 5HF, UK; 2 Medical Research Council Human Genetics Unit, University of Edinburgh, Edinburgh, EH4 2XU, UK; 3 Centre for Cognitive Ageing and Cognitive Epidemiology, University of Edinburgh, Edinburgh, EH8 9XF, UK; 4 Department of Psychology, University of Edinburgh, Edinburgh, EH8 9JZ, UK; 5 Generation Scotland, Centre for Genetics and Experimental Medicine, University of Edinburgh, Edinburgh, EH4 2XU, UK

**Keywords:** Psychological resilience, coping style, heritability, environment, genetics, Generation Scotland, STRADL

## Abstract

**Background:** Twin studies indicate that genetic and environmental factors contribute to both psychological resilience and coping style, but estimates of their relative molecular and shared environmental contributions are limited. The degree of overlap in the genetic architectures of these traits is also unclear.

**Methods:** Using data from a large population- and family-based cohort Generation Scotland (N = 8,734), we estimated the genetic and shared environmental variance components for resilience, task-, emotion-, and avoidance-oriented coping style in a linear mixed model (LMM). Bivariate LMM analyses were used to estimate the genetic correlations between these traits. Resilience and coping style were measured using the Brief Resilience Scale and Coping Inventory for Stressful Situations, respectively.

**Results:** The greatest proportion of the phenotypic variance in resilience remained unexplained, although significant contributions from common genetic variants and family-shared environment were found. Both task- and avoidance-oriented coping had significant contributions from common genetic variants, sibling- and couple-shared environments, variance in emotion-oriented coping was attributable to common genetic variants, family- and couple-shared environments. The estimated correlation between resilience and emotion-oriented coping was high for both common-variant-associated genetic effects (r _G_ = -0.79, se = 0.19), and for the additional genetic effects from the pedigree (r _K_ = -0.94, se = 0.30). Genetic correlations between resilience and task- and avoidance-oriented coping did not meet statistical significance.

**Conclusions:** Both genetics and shared environmental effects were major contributing factors to coping style, whilst the variance in resilience remains largely unexplained. Strong genetic overlap between resilience and emotion-oriented coping suggests a relationship whereby genetic factors that increase negative emotionality also lead to decreased resilience. We suggest that genome-wide family-based studies of resilience and coping may help to elucidate tractable methodologies to identify genetic architectures and modifiable environmental risk factors to protect against psychiatric illness, although further work with larger sample sizes is needed.

## Introduction

Despite significant risk for psychopathology, many individuals exhibit better than expected adjustment. This ability to ‘bounce back’ and maintain or regain mental health despite significant risk is referred to as *psychological resilience* ^1– 3^. Resilience has increasingly become a focus of behavioural and medical research ^4– 6^, promoting positive mental health and offering an alternative to ‘deficit’ models of psychopathology ^7^. Whereas resilience refers to positive adaptation in the face of adversity, coping style encompasses cognitive and behavioural strategies used to manage adversity ^8^. Although often used interchangeably, there is a growing body of evidence to suggest that resilience and coping style are conceptually distinct constructs ^9– 12^. However, the underlying biological mechanisms of and genetic similarities between resilience and coping are not well understood.

A starting point from which to address whether the observed variation in resilience and coping style is due to environmental or biological factors is to investigate heritability. Heritability is broadly defined as the proportion of phenotypic variation that can be attributed to genetic effects ^13^. Such estimates have been applied to the study of both resilience and coping style in family-based twin studies. To illustrate, in defining resilience as the residual for positive affect after controlling for social and interpersonal stressors, a twin study by Boardman and colleagues ^14^ found resilience was significantly more heritable among men (52%) than women (38%). Similarly, another twin study found modest heritability estimates (24–49%) on measures of well-being and mental health which were indirectly related to resilience ^15^. A longitudinal twin study ^4^ estimated resilience as the residual between actual and predicted psychiatric symptoms, based on the total number of stressful life events an individual has experienced, and found moderate heritability estimates at both waves of assessment approximately five years apart (~31%). However, it is important to note that wide discrepancies in both the definition and measurement of resilience preclude accurate comparison of these results ^5^.

Several well-validated questionnaires have been developed to measure coping style ^16– 18^ which focus on task-, emotion-, and avoidance-oriented coping styles. Emotion-oriented coping is characterised by the regulation of distressing emotions, whereas task-oriented coping denotes purposeful efforts aimed at problem solving ^19^. Avoidance-oriented coping is defined by behaviours aimed at avoiding difficult circumstances ^16^. Evidence from twin studies suggests that coping style may be genetically *and* environmentally mediated. For example, a twin study ^20^ found that whereas task-and emotion-oriented coping were modestly heritable (17–20%), avoidance-oriented coping was entirely determined by environmental factors. The majority of the variance in coping style was attributable to non-shared environmental influences. Furthermore, Kozak *et al.* ^21^ found modest genetic influences to the variation seen in each coping style (33–39%), in addition to substantial environmental variance. Kendler *et al.* ^22^ found that approximately 30% of the total variance in the coping styles of ‘turning to others’ and ‘problem solving’ was attributable to genetic effects. Interestingly, however, the variability in the use of ‘denial’ coping styles was entirely accounted for by environmental influences.

Pedigree-based estimates such as those described above measure heritability by comparing the observed phenotypic similarity to the expected genetic resemblance between relatives, and as such, do not require information on the inheritance of individual genetic loci ^23, 24^. However, recent methodological developments in Genome-Wide Association Studies (GWAS) offer an alternative to pedigree-based heritability estimates through molecular estimates in unrelated samples via Single Nucleotide Polymorphisms (SNPs) ^25, 26^. Specifically, this method decomposes the phenotypic variance of a trait into that attributable to common genetic variants (SNPs) and that attributable to the entire genome to estimate narrow-sense heritability ( $h_{n}^{2}$). However, when restricting heritability estimates to include only GWAS significant loci, they only account for a minute proportion of the heritability estimated from twin or pedigree studies, a discrepancy which underlies the ‘‘missing heritability problem’’ ^26, 27^. Yang and colleagues ^28^ have subsequently found that variation in complex traits (such as resilience and coping) are likely resultant from a large number of common variants with effect sizes too small to pass the stringent threshold of GWAS, indicating they are the result of polygenic inheritance. Elaborating on these findings, Zaitlen and colleagues ^29^ have developed a genomic relatedness restricted maximum likelihood (GREML) variance component method that estimates heritability between both related and unrelated individuals, simultaneously. Using genome-wide data this method yields more accurate heritability estimates than other methods because no assumptions are made on the extent of genetic similarity, dominance effects, or epistasis ^29, 30^. Specifically, using the GREML method, heritability estimates have been found to lie between those estimated from pedigree- and GWAS-based studies, and have been considered as a lower limit for the former and an upper limit for the latter ^29, 31^. Furthermore, GREML methods enable researchers to disentangle the differential contributions of molecular and non-additive genetic effects to phenotypic variance whilst simultaneously modelling environmental effects ^29^ which overcomes the caveats of both pedigree- and GWAS-based heritability estimates.

Studies seeking to identify the genetic and environmental contributions to resilience and coping style are an important starting point from which to build an understanding of their aetiology in addition to identifying treatment strategies focussing on primary prevention which may have significant impacts on mental health conditions. In this study, we sought to partition the phenotypic variation of resilience and coping style into their genetic and shared environment components using GREML methods in a family-based genotyped cohort; Generation Scotland: Scottish Family Health Study (GS:SFHS). We drew on the diverse familial relationships within the sample to estimate both molecular and pedigree genetic effects and the contribution of early shared family environment and recent shared environment by analysing family members/siblings and couples respectively. Furthermore, as resilience has been found to be positively correlated with task-oriented coping, and negatively with indices of emotion- and avoidance-oriented coping ^1, 32, 33^, we extended our methods to bivariate analysis to investigate whether these traits have significant overlapping genetic architectures using genetic correlation.

## Methods

### Generation Scotland: Scottish Family Health Study

The Generation Scotland: Scottish Family Health Study (GS:SHFS) ^34^ is a family-based population cohort recruited from General Practitioners’ practices throughout Scotland between 2006 and 2011. Individuals were eligible for participation if they were aged above 18 years and had at least one first-degree relative also willing to participate. A total of 5,628 families (n = 19,200) spanning up to three generations were recruited. In 2014, GS:SFHS participants were re-contacted and asked to take part in a follow-up study of mental health and resilience ^35^. A total of 9,618 participants provided useable re-contact data – a retention rate of 45% - and represent the participants included in the current study. Full cohort details and recruitment procedures for baseline and re-contact are described elsewhere ^34– 36^. All components of GS:SFHS, including its protocol and written study materials have received national ethical approval from the NHS Tayside Committee on Research Ethics (reference 05/s1401/89).

### Genotyping and quality control procedures

At baseline, blood and salivary DNA samples were collected, stored, and genotyped at the Wellcome Trust Clinical Research Facility, Edinburgh. Genome-wide genotype data were generated using the Illumina HumanOmniExpressExome-8 v1.0 DNA Analysis BeadChip (San Diego, CA, USA) and Infinium chemistry ^37^. The details and procedures for DNA extraction and genotyping have been reported extensively elsewhere ^38, 39^. Population outliers were removed from the sample ^40^. Quality control of genotyped SNPs used inclusion thresholds: call rate ≥98%, missing SNPs per individual ≤2%, Hardy-Weinberg equilibrium p>1×10 ^-6^, and minor allele frequency >1%. In total, 561,125 autosomal SNPs for 8,734 individuals remained and were used in subsequent analysis. Multidimensional scaling (MDS) components were created according to the ENIGMA 1000 genomes protocol ^41^ in the software package PLINK ^42^ (version 1.9).

### Resilience, coping style and neuroticism

Psychological resilience was assessed at re-contact using the Brief Resilience Scale (BRS) ^1^, a self-report questionnaire assessing an individual’s ability to ‘bounce back’ or recover from stress. The BRS consists of six statements (e.g., “I usually come through difficult times with little trouble”) answered on a five-point scale from “Strongly Disagree” to “Strongly Agree”. After reverse coding of even-numbered questions, a total resilience score was calculated by computing the mean of six questions. The BRS has been found to have a one-factor structure, demonstrating good internal consistency (Cronbach’s alpha = 0.80–0.91) and test-retest reliability of 0.69 for one month and 0.62 for three months ^1^.

The Coping Inventory for Stressful Situations (CISS) ^43^ was completed at re-contact. The CISS is a 48-item self-report questionnaire in which responders indicate how much they engage in various coping activities “when under stress”, on a five-point scale from (1) ‘Not at all’ to (5) ‘Very much’. Scores are summed over three 16-item sub-scales scales measuring task-oriented (e.g. “when under stress I focus on the problem and see how I can solve it”), emotion-oriented (e.g., “when under stress I blame myself for having gotten into this situation”) and avoidance-oriented (e.g., “when under stress I take time off and get away from the situation”) coping styles. The CISS has proven a robust measure of assessing situation-specific coping strategies, with a stable factor structure, high internal reliability and construct validity ^16^.

## Statistical analysis

### Partitioning of phenotypic variation

Using a recently developed variance component methodology based on the GREML framework, we calculated the contributions of genetic and shared environmental components to the phenotypic variance in resilience, task-, emotion-, and avoidance-oriented coping. Building on the work of Zaitlen *et al.* ^29^, we utilised a new method introduced by Xia *et al.* ^44^ to simultaneously estimate $h_{g}^{2}$ (the proportion of additive genetic variance contributed by common genetic variants over the total phenotypic variance: SNP heritability), $h_{p}^{2}$ (the proportion of additional additive genetic variance attributable by pedigree associated variation), $h_{n}^{2}$ (the proportion of phenotypic variance contributed by all additive genetic variance: narrow-sense heritability), in addition to three shared environmental components. This method has been demonstrated to reliably estimate $h_{n}^{2}$ in related samples overcoming possible inflating and confounding effects within family-based cohorts ^29^. Using genome-wide complex trait analyses ( GCTA: version 1.22) ^30^ the genetic and shared environmental contributions to each trait were measured by partitioning the phenotypic variance using linear mixed modelling (LMM) techniques. For each trait, two genomic relationship matrices; **G** (genomic relationship matrix) and **K** (kinship matrix created by modifying G using a threshold of 0.05 for pairwise relatedness) ^29, 44^, and three environment relationship matrices, **F** (early shared environmental matrix representing nuclear-family-member relationships), **S** (early shared environmental matrix representing full-sibling relationships) and **C** (recent shared environmental matrix representing couple/spousal relationships) ^44^, were fitted separately or simultaneously in LMM. The corresponding variance components $h_{g}^{2}$ (common-variant-associated genetic effect, represented in **G**), $h_{p}^{2}$ (additional genetic effect from pedigree, represented in **K**), $e_{f}^{2}$ (environmental effect from nuclear family, represented in **F**), $e_{s}^{2}$ (environmental effect from full sibling relationship, represented in **S**) and $e_{c}^{2}$ (environmental effect from couple relationship, represented in **C**) were estimated using LMM and their significance tested using likelihood ratio tests (LRT). Age, sex and four MDS components were included in each LMM as fixed effects. Details on the construction of the variance-covariance matrices can be found in the Supplementary File 1.

The initial model was a full model comprising of all genetic and environmental components. However, previous studies ^44, 45^ suggest that variance estimates may be confounded due to correlations between components. To overcome this issue, a backward stepwise model selection was employed. LRT tests were conducted to test the significance of each variance component, which were removed sequentially if they failed to obtain significance (α = 5%) and had the highest *p*-value. This process was repeated until all the remaining components were significant. This method is described in more detail elsewhere ^44, 45^.

To simplify model descriptions, the following codes were used: - e.g. ‘ **GKFSC**’ denotes a full model whereby all five matrices were fitted as random effects simultaneously whereas ‘ **GFC**’ represents a model in which only the genomic relationship matrix, the environmental matrix representing nuclear-family-member relationships and the environmental matrix representing couple relationships were fitted simultaneously.

### Genetic correlation and bivariate heritability

Genetic correlations between resilience and each coping style were calculated to examine potential overlapping genetic architectures. Specifically, bivariate GREML analysis in GCTA ^30, 46^ was conducted to estimate the correlation between pairs of traits for common genetic variants ( *r __G__*), and the pedigree associated genetics ( *r __K__*) simultaneously. These models were controlled for age, sex, and four MDS components. The significance of each genetic correlation was estimated using the LRT.

## Results

Among the 8,734 participants with genome-wide genotyped data, we recognised 655 couple pairs, 1,925 full sibling pairs and 4,508 nuclear families (minimum two individuals). The number of non-zero elements of the **KFSC** matrices for whom genotypic and phenotypic information are available for each trait are shown in the Supplementary Table 1. The mean age of the sample was 56.36 years (SD = 13.15), and 5,403 (62%) were female. Demographic details of these individuals are presented within Supplementary Table 2.

To determine if the traits in this study had sufficient discriminant validity to warrant independent investigation, phenotypic correlations were calculated between resilience and each coping style. The results from age-, and sex-adjusted Pearson correlations are presented in Table 1. Such correlations suggest that whilst these traits are related ( *r* = -0.52 to 0.36), their covariance is sufficiently modest to consider them partially independent variables in our analyses.

**Table 1.** Age-, and sex-adjusted phenotypic Pearson correlations.

	Resilience	ToC	EoC	AoC
Resilience	-			
ToC	0.36 (0.01)	-		
EoC	-0.52 (0.01)	-0.17 (0.01)	-	
AoC	-0.05 (0.01)	0.27 (0.01)	0.30 (0.01)	-

Abbreviations: ToC, Task-oriented coping style; EoC, Emotion-oriented coping style; AoC, Avoidance-oriented coping style All correlations were significant at *p* < 0.01. Values in parentheses represent standard errors

### Full model partitioning phenotypic variation into genetic and shared environmental components

A full model was first employed to partition the phenotypic variation of each trait into five potential sources of influence by modelling two genetic components ( **G** and **K**) alongside three environmental components representing the family, sibling, and couple effects ( **F, S, C**). Specifically, we modelled the effects of additive genetic effects from common variants ( ${\text{h}}_{\text{g}}^{\text{2}}$), additional genetic effects associated with the pedigree ( ${\text{h}}_{\text{p}}^{\text{2}}$), early shared environmental effect shared by nuclear family members ( ${\text{e}}_{\text{f}}^{\text{2}}$), early shared environmental effects shared between full siblings ( ${\text{e}}_{\text{s}}^{\text{2}}$), and recent shared environmental effects shared between spouses ( ${\text{e}}_{\text{c}}^{\text{2}}$). The results of these full models are presented in Table 2 and within Supplementary Table 3. As illustrated in Table 2, neither the genetic or shared environmental components for resilience were statistically significant in the full model. However, in comparison to a reduced model, which does not account for any environmental effects (the **GK** model), the full model obtained lower estimates of genetic variance which suggests that the full model effectively reduced confounding environmental effects when calculating heritability estimates ( Table 2).

**Table 2.** Age-, sex-, and population stratification ^a^-adjusted variance component analyses results for Resilience, ToC, EoC, and AoC.

				G (common-variant associated genetic)	K (pedigree- associated genetic)	F (Nuclear family)	S (Full sibling)	C (Couple)
Trait	n	Model description		$h_{G}^{\text{2}}\text{ (SE)}$	$h_{K}^{\text{2}}\text{ (SE)}$	$e_{F}^{\text{2}}\text{ (SE)}$	$e_{S}^{\text{2}}\text{ (SE)}$	$e_{C}^{\text{2}}\text{ (SE)}$
Resilience	8555	Genetics only	GK	**0.08 (0.04)**	0.06 (0.05)			
		Full	GKFSC	0.06 (0.04)	0.00 (0.12)	0.05 (0.06)	0.00 (0.03)	0.01 (0.07)
		Backward selection	GF	**0.06 (0.04)**		**0.05 (0.02)**		
ToC	8170	Genetics only	GK	**0.12 (0.05)**	**0.13 (0.06)**			
		Full	GKFSC	**0.11 (0.05)**	0.02 (0.13)	0.03 (0.06)	**0.08 (0.04)**	**0.16 (0.07)**
		Backward selection	GSC	**0.14 (0.03)**			**0.10 (0.03)**	**0.18 (0.04)**
EoC	8306	Genetics only	GK	**0.14 (0.04)**	**0.10 (0.06)**			
		Full	GKFSC	**0.14 (0.04)**	0.03 (0.12)	0.04 (0.06)	0.00 (0.03)	**0.14 (0.07)**
		Backward selection	GFC	**0.15 (0.04)**		**0.05 (0.03)**		**0.14 (0.05)**
AoC	8248	Genetics only	GK	**0.14 (0.04)**	**0.09 (0.06)**			
		Full	GKFSC	**0.12 (0.04)**	0.00 (0.13)	0.03 (0.06)	**0.05 (0.03)**	**0.14 (0.07)**
		Backward selection	GSC	**0.15 (0.03)**			**0.07 (0.03)**	**0.18 (0.04)**

^a^ first four MDS components Variance component analyses were performed on Resilience, ToC, EoC, and AoC using the genetic model (GK), the model accounting for both genetic and three environmental effects (the full model), and the most parsimonious model selected by backward selection. Abbreviations: ToC, Task-oriented Coping; EoC, Emotion-oriented Coping; AoC, Avoidance-oriented coping N.B. text in **bold** indicates significant LRT at *p*< 0.05 (one-tailed). Values in parentheses represent standard errors.

For task-oriented coping, the full model estimated that 11% (S.E. = 0.05, *p* = 0.006) of the phenotypic variance was attributable to common genetic variants ( **G**). The pedigree-associated genetic component ( **K**) of this model was not significant, and so the proportion of total additive genetic determination (narrow-sense heritability: $h_{n}^{2}$ = $h_{g}^{2}$ + $h_{p}^{2}$) was resultant from the effect of common-variant associated genetics only ( **G**). Of the three shared-environmental components, both sibling- ( $e_{s}^{2}$ = 0.08, SE = 0.04, *p* = 0.019) and couple-shared ( $e_{c}^{2}$ = 0.16, SE = 0.07, *p* < 0.001) environmental effects met statistical significance. For emotion-oriented coping, 14% (S.E. = 0.04, *p* < 0.001) of its phenotypic variance was determined by common genetic variants, and 14% (S.E. = 0.07, *p* = 0.002) was resultant from couple-shared environmental effects. The environmental effects shared between nuclear family members and full-siblings were not significant. For avoidance-oriented coping, 12% (S.E. = 0.04, *p* = 0.002) of its phenotypic variance was attributable to common genetic variants ( **G**). Significant effects from sibling- ( $e_{s}^{2}$ = 0.05, SE = 0.03, *p* = 0.049) and couple-shared ( $e_{c}^{2}$ = 0.14, SE = 0.07, *p* < 0.001) environment were also found. These results are illustrated in Table 2, and within Supplementary Table 3.

### Backward stepwise model selection to identify major genetic/familial-environmental contributors

Previous research has demonstrated that although the full model can account for all five variance components, it may have difficulty in separating and distinguishing major and minor contributors to the phenotypic variance of a given trait ^44^. To overcome such an issue, we applied stepwise model selection ^44, 45^ to identify major contributors to phenotypic variation in resilience, task-, emotion-, and avoidance-oriented coping.

**Figure 1.**
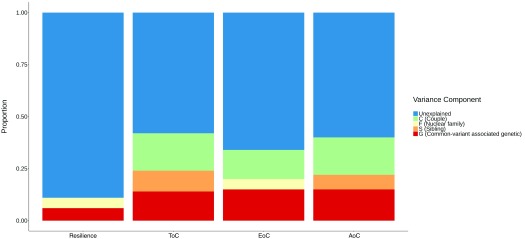
Sources of phenotypic variance and the proportion of variance they explained in the most parsimonious backward stepwise selection models for resilience, task-, emotion-, and avoidance-oriented coping styles. Abbreviations: ToC, Task-oriented Coping; EoC, Emotion-oriented Coping; AoC, Avoidance-oriented Coping.

Using backward stepwise selection for resilience, only the common-variant associated genetic component and shared nuclear-family component were retained in the final model (the **GF** model as shown in Table 2 and Figure 1). Common genetic variants ( **G**) explained 6% (S.E. = 0.04, *p*= 0.041) of the phenotypic variation in resilience and family-shared environmental ( **F**) effects explained 5% (S.E. = 0.02, *p* = 0.020). Using the same methodology, 14% of the variance in task-oriented coping was explained by common-variant associated genetics ( **G**: S.E. = 0.03, *p* < 0.001). Furthermore, 10% of the variance was explained by sibling-shared environmental effects ( **S**: S.E. = 0.03, *p* < 0.001), and a further 18% of the variance was explained by couple-shared environmental effects ( **C**: S.E. = 0.04, *p* < 0.001). Similar patterns were found in avoidance-oriented coping with 15% of the variance explained by common-variant associated genetics ( **G**: S.E. = 0.03, *p* < 0.001), 7% explained by sibling-shared environmental effects ( **S**: S.E. = 0.03, *p* = 0.006), and 18% of the variance explained by couple-shared environmental effects ( **C**: S.E. = 0.04, *p* < 0.001). In examining emotion-oriented coping, it was found that common genetic ( **G** = 0.15, S.E. = 0.04, *p* < 0.001), family-shared ( **F** = 0.05, S.E. = 0.03, *p* = 0.027) and couple-shared environmental effects ( **C** = 0.14, S.E. = 0.05, *p* = 0.002) were most likely to account for phenotypic variance ( Table 2 and Figure 1). These models are presented fully in the Supplementary materials.

### Genetic Correlations

Estimates of common-variant associated (r __G__) and pedigree associated (r __K__) genetic correlations are reported in Table 3, to identify potential overlapping genetic architectures between resilience, task-, emotion-, and avoidance-oriented coping. As no consistent environmental effects were identified in the models above, we explored only the genetic correlations between our traits. Specifically, as there were no consistent environmental contributions across the four traits, and because **K** in the **GK** models captured a mixture of both pedigree associated genetic and environmental effects, we have estimated the genetic correlations for **G** and **K** only. As each trait still has contributions from **K** (based on the full models, above), we will examine this effect to prevent inflation in our **G** estimates ^44, 45^. The genetic correlations between the four traits are illustrated in Table 3. The estimate of the common-variant associated genetic correlation (r __G__) between resilience and task-oriented coping was .51 (S.E. = 0.26, *p* = 0.063). The correlation between resilience and task-oriented coping explained by additional genetic variation associated with pedigree (r __K__) was 0.20 (S.E. = 0.43, *p* = 0.341), although this estimate is potentially influenced by the effects of shared-environment. High genetic overlap was found between resilience and emotion-oriented coping: r __G__ = -0.79 (SE = 0.19, *p* = 0.002), r __K__ = -0.94 (SE = 0.30, *p* = 0.033); and moderate genetic overlap was found between resilience and avoidance-oriented coping: r __G__ = -0.24 (SE = 0.30, *p* = 0.207), r __K__ = 0.36 (SE = 0.54, *p* = 0.237).

**Table 3.** Age-, sex-, and population stratification ^a^-adjusted bivariate GCTA estimates of genetic correlation.

	Resilience	ToC	EoC	AoC
Resilience	-	0.20 (0.43)	**-0.94 (0.30)**	0.36 (0.54)
ToC	0.51 (0.26)	-	-0.46 (0.34)	-0.08 (0.38)
EoC	**-0.79 (0.19)**	-0.05 (0.25)	-	-0.42 (0.54)
AoC	-0.24 (0.30)	**0.48 (0.24)**	**0.60 (0.21)**	-

^a^ first four MDS components Abbreviations: ToC, Task-oriented Coping; EoC, Emotion-oriented Coping; AoC, Avoidance-oriented Coping N.B. text in **bold** indicates significant LRT at *p* < 0.05 (one-tailed). Values in parentheses represent standard errors The genetic correlations between traits resultant from common genetic variance (r _G_) are shown on the lower diagonal; the upper diagonal shows the genetic correlations between traits associated with the pedigree (r _K_)

## Discussion

Here, we report a novel study examining the genetic and environmental contributions to resilience and coping style in GS:SFHS, a population- and family-based cohort comprising close and distant relatives with genome-wide genotyped data. Using a recently developed variance component methodology ^44^, we showed that common variant associated genetics and family-shared environmental effects moderately contribute to psychological resilience, although 89% of its phenotypic variance remained unexplained. We also found that each coping style had substantial genetic ( ^~^20%) and shared environmental (20–30%) contributions. Furthermore, we found large genetic correlations between resilience and emotion-oriented coping for both common-variant associated (r _G_ = -0.79) and pedigree-associated (r __K__ = -0.94) genetic effects, which suggests that genetic effects have a shared influence on both traits but in opposite directions. Such findings indicate that genetic factors that increase negative emotionality lead to reduced psychological resilience, which mirror previous reports that which suggest resilience and traits characterised by negative emotionality provide partially separate mechanisms to reduce and increase susceptibility to psychopathology, respectively ^47^.

Although the majority of phenotypic variance in resilience remained unexplained in this study, (small) significant contributions from common variant associated genetic and family-shared environmental effects were found. Within our study, the family effect represents the ‘nuclear’ family, an early environmental influence associated with living in the same family group. It has been found that children with poor familial relationships are more likely to develop psychopathology in later life ^48^, whereas positive family relationships have been found to prevent negative mental health outcomes in ‘at-risk’ children ^49^, which supports our finding that family-shared environment influences resilience. Behavioural genetics studies suggest that positive familial relationships enable an individual to regulate their behaviour and emotions to perceive their environment as manageable, no matter how challenging ^50, 51^. Furthermore, previous studies have demonstrated that strong familial attachments in childhood have long-lasting impacts on resilience and general well-being in later life ^52^, which is important within the context of this study which examined adults who may no longer be living within the ‘nuclear’ family environment, but whose effects are still apparent.

In examining both genetic and environmental effects simultaneously, we also detected an almost equal contribution from common-variant associated genetic and couple-shared environment effects for all three coping styles. The couple effect reflects the current environment shared between spouses in adulthood, which contrasts with both the full sibling and nuclear family effect which reflects the influence of earlier shared environments. During stressful circumstances, the support of a spouse (living in the same household) is more likely to be sought than support from closely related family members (living in a different household) ^53^. The major contribution of couple-shared environment to coping could potentially capture the effects of assortative mating ^54^, and other factors leading to spousal similarity. However, this effect may also be explained by couples learning from each other and adapting their coping styles to better face the adversity at hand ^55^. Comparatively less variance was accounted for by sibling- and family-shared environmental effects which may be due to the high correlation between the matrices which could potentially impede model fit and estimation ^45^. Previous simulation of these models ^44^ suggest that true components are detected approximately 80% of the time and so the small sibling- and family-shared environmental effects found could be due to false positives in the model. However, without a larger sample size, it would be difficult to have the power to fully discriminate between these components ^44^, and so we advocate further replication in independent samples.

We also examined the genetic correlations between resilience and each coping style. Our results revealed very high negative correlations between resilience and emotion-oriented coping for both common-variant associated genetic and pedigree-associated genetic components. These findings suggest that there is a strong shared genetic architecture between resilience and emotion-oriented coping whereby genetic factors that increase negative emotionality also lead to decreased resilience. The direction of these findings supports previous research which suggests that individuals high in negative emotionality, and low in resilience are at a greater risk for psychopathology ^1, 32^. We must note, however, that correlations for pedigree-associated genetic components are likely biased due to the influence of shared-environmental effects which may be contained within the pedigree component, or vice versa. Unfortunately, due to a lack of power and model non-convergence, we were unable to report the environmental correlations between these traits. It would be of benefit to further investigate the genetic and environmental correlations between these traits in a larger sample to underpin important differences between the traits. For example, in further investigating the environmental correlations between resilience and coping style, we may be able to determine if having a resilient spouse is associated with a particular coping style.

The narrow-sense heritability estimate ( $h_{n}^{2}$ = $h_{g}^{2}$ + $h_{p}^{2}$, see Supplementary Table 4) found for resilience in the current study was substantially less than broad-sense estimates derived from twin studies ^4, 14, 15^. This is unsurprising given that previous reports suggest that GREML-based estimates provide a lower limit for pedigree-based estimates, and an upper limit for GWAS-based estimates ^29, 31^, although without any existing GWAS data on resilience, it difficult to know this for certain. However, the narrow-sense heritability estimates of task- and emotion-oriented coping in this study were in line with previous reports ^20– 22^. Furthermore, genetic estimates of avoidance-oriented coping were at odds with previously reported heritability estimates which found no genetic effects in avoidance-oriented coping styles ^20, 21^. This may be due to our sample being better powered to detect genetic components of avoidance-oriented coping in comparison to previous twin studies ^20– 22^ which found conflicting results with much smaller samples (n < 1,000). In our analyses, pedigree-associated genetics (which include rarer genetic variants and mutations) showed no significant contribution to any of our four traits. This is a novel finding as previous estimates suggest that for most complex traits over 50% of narrow-sense heritability is attributable to pedigree-associated genetic effects ^29, 44^. This could be because our sample may not have had sufficient power to separate out pedigree effects from shared environmental effects, indicated by our study failing to detect any significant pedigree effects. Alternatively, this study may have been confounded by correlations between components ^44, 45^.

A number of limitations to this study deserve mention. Firstly, we employed a quantitative trait-based measure of resilience, whereas other behavioural genetic studies have found larger genetic effects with both outcome- and process-based approaches ^4, 14, 56^. Specifically, the Brief Resilience Scale assesses an individual’s ability to ‘bounce back’ from adversity and is purposely framed in regards to overcoming negative events ^1^ whereas previous heritability estimates for resilience have predominately focussed on assessing the availability of implicit assets and resources which facilitate resilience ^57, 58^. It is important to make this distinction clear as this difference may underlie the different heritability estimates reported in the literature, and will inevitably hinder comparison between studies and preclude any meta-analysis ^6^. Furthermore, as resilience and coping are related constructs, previous estimates of resilience heritability may have indexed, in part, the role of coping style leading to inflated estimates. Secondly, as the re-contact cohort was a sub-set of the larger GS:SFHS sample, we were constrained by a limited number of participants with a reduced familial structure. Future investigation would greatly benefit from a larger sample size with an increased number of familial relationships to fully disentangle environmental components in the relationship between these traits. Furthermore, although we obtained estimates from effects from common-variant and pedigree-associated genetic, our sample is underpowered to detect small effects ^44^ which would be overcome by a larger sample size, either related (using our methodology) or between two independent datasets (using methods such as LD-score regression ^59, 60^). Finally, there may be other major shared and non-shared environmental effects of each of our traits that are not specifically captured in our analysis. For example, research suggests that resilience may be associated with stressful life events, growing up in adversity, or being raised in care ^61– 65^.

Here, we provide evidence that psychological resilience (quantified by a previously validated ordinal scale), is a heritable trait with a relatively small proportion of its variance explained by genetic factors. Early childhood environment such as that shared by the nuclear family was also found to have a small association with resilience. Task-, emotion- and avoidance-oriented coping styles were found to be moderately heritable, although substantial environmental effects also contributed to their phenotypic variance. Approximately one fifth of the variance in each coping style was attributable to recent environment shared by couples. These results indicate that both genetic and environmental contributors to resilience and coping style need to be considered in future research. Finally, high negative genetic correlations between resilience and emotion-oriented coping suggests that the traits share an overlapping genetic architecture in which genetic factors that increase negative emotionality lead to reduced resilience. This is the first study to date which aimed to disentangle the molecular and environmental components in resilience and coping style, and represents a valuable starting point from which to further elucidate the underlying mechanisms of these traits. We argue that further work with larger samples sizes is necessary to fully delineate the genetic and environmental contributions of these traits, and the relationships between them to identify modifiable protective factors against psychological distress and illness.

## Data availability

Due to the confidential nature of the genetic and phenotypic data provided by participants in this study, it is not possible to publically share the data on which our analyses were based. A phenotype data dictionary from Generation Scotland (GS) is available and can be downloaded. Non-identifiable information from the GS:SFHS cohort is available to researchers in the UK and to international collaborators through application to the GS Access Committee: access@generationscotland.org, with further information available from http://www.ed.ac.uk/generation-scotland. Each application requires the completion of a data and materials transfer agreement, the conditions of which are determined on a case by case basis. GS has Research Tissue Bank status, and the GS Access Committee reviews applications to ensure that they comply with legal requirements, ethics and participant consent.

## Ethical statement

Ethics approval for the Generation Scotland study was given by the NHS Tayside committee on research ethics (reference 05/S1401/8).

## Supplementary material

**Supplementary File 1:** Construction of Genetic Relationship Matrices (GRMs).

**Supplementary Table 1:** Number of non-zero off diagonal entries for each trait.

**Supplementary Table 2:** Descriptive statistics of the current cohort.

**Supplementary Table 3:** Narrow-sense heritability estimates for the four traits examined.

**Supplementary Table 4:** Full variance component analysis.
