# Supplementary material for: Genetic and environmental contributions to psychological resilience and coping
Source: Wellcome Open Res. 2018 Feb 15;3:12. [Version 1] doi: 10.12688/wellcomeopenres.13854.1 (PMC6192447; doi:10.12688/wellcomeopenres.13854.1)
Supplement: Supplementary file 1 [file wellcomeopenres-3-15058-s0000.tgz › 779f0eae-0974-4040-b1ac-0bb7c346a67c.pdf]

Construction of Genetic Relationship Matrices (GRMs)

Two GRMs were fitted using the method created by Zaitlen et al (2013). The first GRM comprised of pairwise relationship coefficients of all individuals in the sample. The second GRM included off-diagonal elements of pairs of individuals who has a relationship coefficient < 0.05 set to 0. Assuming inbreeding had not taken place, the second GRM excluded pairs of individuals with a most recent common ancestor of approximately four generations distant. This method has been found to account for potential upward biases due to excessive relationships which allows for the inclusion of closely and distantly related individuals in genetic analyses

Constructing variation-covariance matrices representing different source of variation

G: Genomic relationship matrix: The genetic relationships between individuals were calculated in GCTA using the following formula:

$$Ajk = \frac{1}{N}\sum_{i=1}^{N} \frac{(xij - 2pi)(xik - 2pi)}{2pi(1 - pi)}$$

Yang et al. (2011)

in which i represents a SNP, x is the allele count of the minor allele for individual j or k at i. p_i is the minor allele frequency of SNP i , and N is the total number of SNPs. This matrix was created in GCTA.

K: Kinship relationship matrix: K was calculated by the modification of the G matrix. Relationship co-efficient values less than or equal to 0.05 in the G matrix were set to 0, as this threshold separates closely and distantly related individuals (Zaitlen et al, 2013).

F,S,C: Environmental relationship matrices: To represent different shared environmental effects, familial relationship matrices were designed. Each was created by making an N x N matrix in which all entries were set to 0 and all diagonal entries set to 1. Off-diagonal entries, too, were set to 1 if two families shared the environment of interest. A total of three environmental relationship matrices were created; **F** represents the shared environment of a nuclear family living within the same household, **S** represents the sibling environment, and **C** represents the couple environment.

Estimating the phenotypic variance explained by different source of variation.

The genomic and environment relationship matrices described above were selectively jointly fitted in a Linear Mixed Model (LMM) implemented in GCTA. The models analysed included all fixed effects and subsets of random effects in the full model:

$$Y = Xb + g_g + g_{kin} + e_f + e_s + e_c + \varepsilon$$

In which Y is a phenotype vector, *b* is a vector of covariates fitted as fixed effects (age, sex, and four principal components derived from the genome-wide genomic relationship matrix). g_g and $g_{kin}$are random genetic effects from SNPs and the extra random genetic effect from the pedigree, respectively. e_f, e_s, e_c represent random environmental effects shared by nuclear family members, full-siblings and couples, respectively. For simplicity, the following codes were used to represent the matrices fitted: for example, '***GKFSC***' was the full model fitting all five matrices as random effects simultaneously, whereas '***GFC***' represents a model in which the genomic relationship matrix, nuclear family and couple environment matrices were simultaneously fitted. The proportion of variance explained by individual component was estimated using REML and tested using Log-likelihood Ratio Test (LRT) in GCTA.

REFERENCES

Zaitlen, N., Kraft, P., Patterson, N., Pasaniuc, B., Bhatia, G., Pollack, S., et al. (2013). Using extended genealogy to estimate components of heritability for 23 quantitative and dichotomous traits. *PloS Genet;* 9(5): e1003520.

Yang, J., Lee, S.H., Goddard, M.E., Visscher, P.M. (2011). GCTA: a tool for genome-wide complex trait analysis. *Am J Hum Genet*, 88(1), 76-82.
