# Supplementary material for: Genetic and environmental contributions to psychological resilience and coping
Source: Wellcome Open Res. 2018 Feb 15;3:12. [Version 1] doi: 10.12688/wellcomeopenres.13854.1 (PMC6192447; doi:10.12688/wellcomeopenres.13854.1)
Supplement: Supplementary file 2 [file wellcomeopenres-3-15058-s0001.tgz › b5643b61-1955-40a1-9d2a-84df7a522be7.pdf]

Supplementary Table 1

Number of non-zero off diagonal entries in the lower triangular part of all variance component matrices

Matrix	Number of non-zero off diagonal entries				
	Full sample	Resilience	ToC	EoC	AoC
K - pedigree associated genetics	6,015	5,810	5,314	5,501	5,439
F – nuclear family	4,508	4,340	3,982	4,115	4,057
C – couple	655	633	574	588	1,753
S -sibling	1,925	1,863	1,733	1,774	574

Abbreviations: ToC; Task-oriented Coping; EoC, Emotion-oriented Coping; AoC, Avoidance-oriented Coping
N.B. As all entries in the G (common-variant associated genetics) matrix are non-zero this matrix is not presented here.
