# Supplementary material for: Genetic and environmental contributions to psychological resilience and coping
Source: Wellcome Open Res. 2018 Feb 15;3:12. [Version 1] doi: 10.12688/wellcomeopenres.13854.1 (PMC6192447; doi:10.12688/wellcomeopenres.13854.1)
Supplement: Supplementary file 3 [file wellcomeopenres-3-15058-s0002.tgz › 72e17218-42e4-481a-9828-a40f905f104b.pdf]

Supplementary Table 2

Descriptive data from the individuals in this sample (n = 8,734)

Variable	n	Mean (SD)	n (%)	Median	Range
Age	8734	56.36 (13.15)		59	22 – 100
Sex (Female)	8734		5,403 (62)		
Resilience	8557	3.56 (0.80)		3.67	1- 5
ToC	8172	54.38 (12.21)		56	16 – 80
EoC	8308	37.62 (12.55)		37	16 – 80
AoC	8250	39.43 (10.49)		40	16 – 80

Abbreviations: ToC, Task-oriented coping; EoC, Emotion- oriented coping; AoC, Avoidance-oriented coping
