# Supplementary material for: Genetic and environmental contributions to psychological resilience and coping
Source: Wellcome Open Res. 2018 Feb 15;3:12. [Version 1] doi: 10.12688/wellcomeopenres.13854.1 (PMC6192447; doi:10.12688/wellcomeopenres.13854.1)
Supplement: Supplementary file 4 [file wellcomeopenres-3-15058-s0003.tgz › 65c808b9-44c7-49ea-b1b8-4171b6de8c0d.pdf]

Supplementary Table 3

Age-, sex-, and population stratification[a]-adjusted univariate GCTA estimates of narrow-sense heritability

	n	V_G (SE)	V_K (SE)	V_e(SE)	V_p (SE)	V_G/V_P (SE)	V_K/V_P(SE)	h_n^2
Resilience	8555	0.05 (0.03)	0.04 (0.03)	0.54 (0.02)	0.63 (0.01)	**0.08 (0.04)**	0.06 (0.05)	**0.14 (0.09)**
ToC	8170	17.26 (6.74)	18.56 (8.32)	112.27 (5.21)	148.08 (2.34)	**0.12 (0.05)**	**0.13 (0.06)**	**0.24 (0.10)**
EoC	8306	19.84 (6.46)	14.41 (7.98)	110.45 (5.04)	144.69 (2.26)	**0.14 (0.04)**	**0.10 (0.06)**	**0.24 (0.10)**
AoC	8248	12.91 (4.26)	8.57 (5.31)	73.75 (3.33)	94.75 (1.49)	**0.14 (0.04)**	0.09 (0.06)	**0.23 (0.10)**

[a] first four principal components

Abbreviations: ToC, Task-oriented Coping; EoC, Emotion-oriented Coping; AoC, Avoidance-oriented coping; V_G, variance associated with additive genetic effect from common variants, V_K, variance associated with the pedigree; V_e, residual variance, V_p, phenotypic variance, V_G/V_P, ratio of additive genetic effect from common variants to phenotypic variance; V_K/V_P, ratio of pedigree variances to phenotypic variance; h_n^2, narrow-sense heritability

N.B. text in **bold** indicates LRTp< 0.05 (one-tailed).
