# Supplementary material for: Genetic and environmental contributions to psychological resilience and coping
Source: Wellcome Open Res. 2018 Feb 15;3:12. [Version 1] doi: 10.12688/wellcomeopenres.13854.1 (PMC6192447; doi:10.12688/wellcomeopenres.13854.1)
Supplement: Supplementary file 5 [file wellcomeopenres-3-15058-s0004.tgz › 4dfb8f69-b28f-4e4e-a1d8-bc00216c0ae0.pdf]

Supplementary Table 4

Age-, sex-, and population stratification[a]-adjusted variance component analyses results for Resilience, ToC, EoC, and AoC

Variable	n	Model	h_g^2 (SE)	h_k^2(SE)	e_f^2(SE)	e_s^2(SE)	e_c^2 (SE)
Resilience	8,555	GK	**0.08 (0.04)**	0.06 (0.05)			
		GKFSC	0.06 (0.04)	0.00 (0.12)	0.05 (0.06)	0.00 (0.03)	0.01 (0.07)
		GFSC	**0.07 (0.04)**		0.05 (0.03)	0.00 (0.03)	0.02 (0.05)
		GFC	**0.07 (0.04)**		0.04 (0.03)		0.04 (0.05)
		GF	**0.06 (0.04)**		**0.05 (0.02)**		
ToC	8,170	GK	**0.12 (0.05)**	**0.13 (0.06)**			
		GKFSC	**0.11 (0.05)**	0.02 (0.13)	0.03 (0.06)	**0.08 (0.04)**	**0.16 (0.07)**
		GFSC	**0.12 (0.04)**		0.03 (0.03)	**0.08 (0.04)**	**0.15 (0.05)**
		GSC	**0.14 (0.03)**			**0.10 (0.03)**	**0.18 (0.04)**
EoC	8,306	GK	**0.14 (0.04)**	**0.10 (0.06)**			
		GKFSC	**0.14 (0.04)**	0.03 (0.12)	0.04 (0.06)	0.00 (0.03)	**0.14 (0.07)**
		GFSC	**0.14 (0.04)**		**0.06 (0.03)**	0.00 (0.03)	**0.13 (0.05)**
		GFC	**0.15 (0.04)**		**0.05 (0.03)**		**0.14 (0.05)**
AoC	8,248	GK	**0.14 (0.04)**	0.09 (0.06)			
		GKFSC	**0.12 (0.04)**	0.00 (0.13)	0.03 (0.06)	**0.05 (0.03)**	**0.14 (0.07)**
		GFSC	**0.13 (0.04)**		0.03 (0.03)	**0.06 (0.03)**	**0.15 (0.05)**
		GSC	**0.15 (0.03)**			**0.07 (0.03)**	**0.18 (0.04)**

[a] first four principal components

Abbreviations: ToC; Task-oriented Coping; EoC, Emotion-oriented Coping; AoC, Avoidance-oriented Coping; h_g^2, common variants -associated genetic effect; h_k^2, pedigree associated genetic effect; e_f^2, nuclear family environmental effect; e_s^2, full sibling environmental effect; e_c^2, couple environmental effect

N.B. Backward stepwise selection was used to select the most parsimonious model for each trait.

Text in **bold** indicates LRTp< 0.05 (one-tailed).
