# Supplementary material for: Genetic and environmental contributions to psychological resilience and coping
Source: Wellcome Open Res. 2018 Feb 15;3:12. [Version 1] doi: 10.12688/wellcomeopenres.13854.1 (PMC6192447; doi:10.12688/wellcomeopenres.13854.1)
Supplement: Peer review history

# Peer review: Referee response for version 1

**DOI:** 10.21956/wellcomeopenres.15058.r34039

**Published:** 2018-10-15

**Authors:** Margaret Gatz

**Affiliations:** 1 Center for Economic and Social Research, University of South California, Los Angeles, CA, USA

In this paper, the authors estimate genetic and shared environmental contributions to resilience and coping, and subsequently the genetic correlations between these phenotypes. They use the Generation Scotland: Scottish Family Health Study, comprised of sibling, couple, and nuclear family dyads, from whom DNA samples have been collected. They simultaneously estimate heritability based on common genetic variants and additional additive genetic variance based on family relatedness, as well as early shared environment based on nuclear family and sibling relationships and recent shared environment based on couple relationships. Finally, a bivariate model simultaneously calculated genetic correlations between pairs of traits based on genetic variance from common genetic variants and estimated from relatedness. The statistical modelling, building on GREML, represents an elegant advance in disentangling genetic and shared environmental contributions where there are a variety of degrees of relatedness.

 The authors measured psychological resilience with the Brief Resilience Scale that assesses self-reported ability to bounce back from stress. Coping was measured with the Coping Inventory for Stressful Situations (CISS) that provides scores for task-oriented, emotion-oriented, and avoidance-oriented coping.

 For resilience, estimated narrow sense heritability based on additive genetic effects was 14%, which is considerably lower than prior reports using a twin design. However, the twin studies used quite different measures of resilience. Estimated heritability for the three coping scales was 23% to 24%. This result is not substantially at odds with two prior reports using a twin design and the same measure. Estimates of genetic effects based on common variants were not statistically significant for resilience and ranged from 11% to 14% for the three coping scales.

 Shared environmental effects were not statistically significant for resilience. For task-oriented coping, 27% of the variance was explained by shared environment, especially couple and to a lesser extent sibling effects. Similarly, for avoidance-oriented coping, 22% of the variance was explained by shared environment, again attributable to couple and sibling effects. For emotion-focused coping, 18% of the variance was explained by shared environment, mainly reflecting couple effects that represent current shared environment (or, as the authors point out, potentially assortative mating). The potential of the statistical modelling method is nicely illustrated by these results.

 The estimated genetic correlation between resilience and emotion-oriented coping was high and negative, whether based on common variants (-.79) or pedigree (-.94). There were positive but not statistically significant genetic correlations between resilience and task-oriented coping. The correlation between resilience and avoidance-oriented coping was negative but not statistically significant when based on common variants and positive but not statistically significant when based on pedigree.

 Unfortunately it was not possible to estimate shared environmental correlations.

 The authors conclude that the genetic factors that are associated with increased negative emotionality are also associated with lower resilience.

 With respect to the implications of this study for understanding coping and resilience, a key issue not introduced by the authors is their choice of a measure of coping. Coping styles are strategies that people use to manage stress and adversity. Typically, problem-focused coping refers to practical ways to address a problem or situation that is causing stress, whereas emotion-focused coping refers to strategies reduce and regulate the distressing emotions arising from the problem or situation. Classic measures of coping include Carver’s COPE and Folkman & Lazarus’s Ways of Coping scale.

 The CISS measure of task-oriented coping parallels problem-focused coping as assessed by other coping scales. The CISS measure of avoidance-oriented coping largely encompasses distraction, including social diversion, somewhat analogous to self-distraction on the Brief COPE. However, the CISS measure of emotion-oriented coping is comprised of items that are largely about self-blame, which would be categorized by other coping scales as ineffectual coping. In contrast, on the COPE or Ways of Coping, emotion-focused coping encompasses positive reappraisal, acceptance, use of emotional support, etc.

 It is necessary to appreciate this point in order to make sense of the findings and to consider translational implications. The authors indirectly acknowledge this problem, in that, in the discussion section, they frame the negative relationship between resilience and emotion-oriented coping as a relationship between reduced resilience and *negative emotionality.* However, there is no explicit mention of the measurement issue, no explicit explanation that the emotion-oriented scale is best understood as a measure of neurotic coping, and no recognition that genetic and environmental contributions to emotion-focused coping as commonly understood are not actually evaluated in this study. The section headed “Resilience, coping style and neuroticism” could be a place to discuss this issue, and to redefine the CISS emotion-focused coping scale as negative emotionality or neurotic coping.

 The data are available through applying to Generation Scotland and completing a data and materials transfer agreement.

I have read this submission. I believe that I have an appropriate level of expertise to confirm that it is of an acceptable scientific standard.

# Peer review: Referee response for version 1

**DOI:** 10.21956/wellcomeopenres.15058.r30897

**Published:** 2018-05-09

**Authors:** Maria Antonietta Stazi

**Affiliations:** 1 Center for Behavioral Sciences and Mental Health, National Institute of Health, Rome, Italy

This study aims to disentangle the genetic and environmental contributions to resilience and coping style in Generation Scotland (GS), a population and family-based cohort with genome-wide genotyped data.

 Psychological resilience was evaluated by the Brief Resilience Scale (BRS), while the coping style was assessed by the Coping Inventory for Stressful Situations (CISS) which measures task-oriented (ToC), emotion-oriented (EoC) and avoidance oriented (AoC) coping styles.

 The contributions of genetic and shared environmental components to the phenotypic variance in resilience, ToC, EoC, and AoC was estimated using a variance component methodology based on the work of Zaitlen (2013) and Xia (2016) which allows to separately and simultaneously estimate, in genotyped family based studies, the heritability (the proportion of phenotypic variance attributable to genetic variance) due to common genetic variants and to pedigree associated variation.

 The authors find that common variant associated genetics and family shared environmental effects moderately contribute to psychological resilience (almost 90% of its phenotypic variance remained unexplained), while the contribution of genetics and family shared environmental is a bit larger in all the coping styles.

 Interesting are the results from the bivariate analysis estimating the genetic correlations between resilience and EoC, where large genetic correlations is found for both common-variant associated and pedigree associated genetic effects, suggesting that genetic effects have a shared influence on both traits in opposite directions: genetic factors that increase negative emotionality lead to reduced psychological resilience.

 The paper frames the objective of the study in the contest of the current literature and reports clearly and quite accurately its various phases.  The study design is appropriate and the work is technically sound. Details of methods are adequate and analysis could be replicated by other researchers.

 As regards the statistical analysis, I’m always a bit uncomfortable with the stepwise model selection procedures and I rather prefer to discuss the results from full models, especially when the study sample is underpowered to detect small effects. Furthermore, results from full models are more suitable to be compared across various studies. I appreciate in this paper the effort to illustrate, also in the supplementary materials, the findings of the various models.

 The authors declare that due to the confidential nature of the genetic and phenotypic data provided by participants in this study, it is not possible to publicly share the data on which the analyses are based. However, non-identifiable information from the Generation Scotland cohort is available to researchers through application to the GS Access Committee.

 The authors claim that psychological resilience and coping styles are moderate heritable traits, with a relatively small proportion of their variances explained by genetic factors (common variant or pedigree) and, with even more less extent,  by environmental family shared factors. They propose that both genetic and environmental contributors to resilience and coping style need to be considered in future research, perhaps in order to shrink the missing  heritability. Certainly this is a good research suggestion, but I would like to see here a bit of discussion or speculation on the huge proportion of phenotypic variance unexplained by the mentioned component: a part from the role of the error, there is any room for debating on the importance of unshared environment (the very personal experience) on the occurrence or prevention of these psychological traits?

 Minor points:

 I suggest to use always K or p to denote the pedigree associated genetic component. It easier for the reader to follow the notation.

 Table 1: are the values in parentheses really the SEs? or the p<0.01?

 Table 3: the last footnote, second last row, first word: variance should be variant.

I have read this submission. I believe that I have an appropriate level of expertise to confirm that it is of an acceptable scientific standard.
